# Hyperactivity Induced by Soluble Amyloid-β Oligomers in the Early Stages of Alzheimer's Disease

**DOI:** 10.3389/fnmol.2020.600084

**Published:** 2021-01-07

**Authors:** Audrey Hector, Jonathan Brouillette

**Affiliations:** Department of Pharmacology and Physiology, Hôpital du Sacré-Cœur de Montréal Research Center, Centre intégré universitaire de santé et de services sociaux du Nord-de-l'Île-de-Montréal (CIUSSS-NIM), Université de Montréal, Montreal, QC, Canada

**Keywords:** amyloid-beta oligomers, hyperactivity, neurodegeneration, memory, epileptiform activity, gamma oscillations, slow wave

## Abstract

Soluble amyloid-beta oligomers (Aβo) start to accumulate in the human brain one to two decades before any clinical symptoms of Alzheimer's disease (AD) and are implicated in synapse loss, one of the best predictors of memory decline that characterize the illness. Cognitive impairment in AD was traditionally thought to result from a reduction in synaptic activity which ultimately induces neurodegeneration. More recent evidence indicates that in the early stages of AD synaptic failure is, at least partly, induced by neuronal hyperactivity rather than hypoactivity. Here, we review the growing body of evidence supporting the implication of soluble Aβo on the induction of neuronal hyperactivity in AD animal models, *in vitro*, and in humans. We then discuss the impact of Aβo-induced hyperactivity on memory performance, cell death, epileptiform activity, gamma oscillations, and slow wave activity. We provide an overview of the cellular and molecular mechanisms that are emerging to explain how Aβo induce neuronal hyperactivity. We conclude by providing an outlook on the impact of hyperactivity for the development of disease-modifying interventions at the onset of AD.

## Introduction

Synapse loss that precedes neuronal death is the strongest predictor of cognitive decline in Alzheimer's disease (AD) (Alzheimer's, 2020). Although we still need to uncover all the cellular and molecular events leading to neurodegeneration in AD, it is well-established that toxic soluble low-molecular-weight amyloid-beta oligomers (Aβo) play an essential role in synapse loss and strongly correlate with the clinical state of AD patients (Selkoe, 2002; Brouillette, 2014). Since soluble Aβo start to accumulate in the brain up to two decades before the appearance of clinical symptoms (Cline et al., 2018), understanding how Aβ pathology disturbs cell functioning and neuronal networks would be exceedingly beneficial to develop novel therapeutic approaches to prevent memory deficits at the onset of AD before neurodegeneration induces irreversible brain damages that drastically compromises the quality of life of the patient.

Aβ peptides are composed of 36–43 amino acids and are produced by the proteolytic cleavage of the transmembrane amyloid precursor protein (APP) by β- and γ-secretases (Haass et al., 2012). Given their hydrolytic properties, Aβ peptides (especially Aβ_1−42_) tend to oligomerize rapidly and dynamically until they form insoluble fibrils that aggregate into plaques. Many Aβ species have been shown to be neurotoxic such as dimers, trimers, tetramers, nonamers, dodecamers, protofibrils, and fibrils (Brouillette, 2014). Whereas some reports have highlighted the neurotoxic effects of particular Aβ intermediates with a defined size and structure, other studies have used mixtures of various Aβ species to measure the global impact of the different species that are found simultaneous in the brain. Although a large amount of studies have consistently reported the deleterious impact of soluble Aβo on synapse function and cognitive performance using different types of Aβ preparations in AD mouse models, *in vitro*, and in humans, the primary events disturbed by Aβo which drive the neurodegenerative process still need to be elucidated.

Cognitive impairment in AD was traditionally assumed to originate from lower synaptic activity that eventually lead to neurodegeneration. Multiple lines of evidence now indicate that, particularly in the early stages of AD, synapse dysfunction and loss are first induced by neuronal hyperactivity rather than hypoactivity (Busche et al., 2012, 2015a). Over the past few years, a growing body of evidence has highlighted the major role of soluble Aβo in the induction of neuronal hyperactivity at the onset of AD. Based on AD animal models, *in vitro* experiments and human studies, Aβo-induced neuronal hyperactivity has emerged as an early functional hallmark of AD which triggers synaptic failure, memory dysfunction, epileptiform activity, and neurodegeneration.

## Neuronal Network Hyperactivity in Humans

In human, brain activity can be investigated by functional magnetic resonance imaging (fMRI), positron emission tomography (PET), single-photon emission computed tomography (SPECT), and electroencephalogram (EEG) recordings at resting state or while executing a cognitive task. Hippocampal hyperactivation has been detected by fMRI during memory-encoding tasks in people with mild cognitive impairment (MCI), a prodromal stage of AD, as well as in pre-symptomatic individuals carrying the E280A presenilin-1 (PS1) mutation, the most common cause of early-onset familial AD (Dickerson et al., 2005; Celone et al., 2006; Quiroz et al., 2010; Bakker et al., 2012; Sepulveda-Falla et al., 2012) (Table 1). Higher hippocampal activation was also observed before any clinical symptoms in carriers of the APOE4 allele, the most important genetic risk factor for late-onset sporadic AD (Bookheimer et al., 2000; Trivedi et al., 2008; Filippini et al., 2009; Kunz et al., 2015).

**Table 1:** Neuronal hyperactivity in humans, AD animal models, and cell cultures.

**Humans, animal models, and cell cultures**		**Periods of neuronal hyperactivity**	**Brain regions**	**References**
Humans	MCI	Prodromal AD	Hippocampus	Dickerson et al., 2005; Celone et al., 2006; Bakker et al., 2012
	PS1 E280A	Pre-symptomatic AD	Hippocampus	Quiroz et al., 2010; Sepulveda-Falla et al., 2012
	APOE4	Before clinical symptoms of AD	Hippocampus	Bookheimer et al., 2000; Trivedi et al., 2008; Filippini et al., 2009; Kunz et al., 2015
Animal models	APP23 × PS45	1–2 mo old	Hippocampus and cortex	Busche et al., 2008, 2012
	APP23, APPPS1	18 mo old	Frontal cortex	Maier et al., 2014
	hAPP-J20	4–6 mo old	Parietal cortex	Sanchez et al., 2012
	3 × Tg-AD	8–10 mo old	Cortex	Nygaard et al., 2015
	APPswe/PS1D9	6–7 mo old	Visual cortex	Rudinskiy et al., 2012
	Aβ-containing AD brain extracts, Aβ dimers	Immediately after Aβ injection in WT mice	CA1 area	Busche et al., 2012; Zott et al., 2019
Cell cultures	Aβ_25−35_	Immediately after Aβ application	Rat hippocampal cultures and slices	Brorson et al., 1995
	Aβ_1−42_ oligomers	24 h after Aβ application	Mouse hippocampal cultures	Ciccone et al., 2019
	Tg2576 mice	Embryos (cultures) and 3 mo old (slices)	Hippocampal cultures and slices	Ciccone et al., 2019
	Endogenously released human Aβ	1 h after inhibition of neprilysin	Rat hippocampal cultures and slices	Abramov et al., 2009
	Aβ_1−40_ monomers and dimers	15 min after Aβ application	Hippocampal cultures and slices	Fogel et al., 2014
	Aβ-containing AD brain extracts, Aβ dimers	Immediately after Aβ application	Mouse hippocampal slices	Zott et al., 2019
	PS1ΔE9, PS1M146V, APPswedish mutants	5–6 weeks of differentiation	hiPSC-derived neurons	Park et al., 2018; Ghatak et al., 2019

As the disease progresses, neuronal networks gradually switch to hypoactivity in AD during memory encoding (Celone et al., 2006; Persson et al., 2008; Reiman et al., 2012). Although there is currently many different compounds such as the Pittsburgh Compound B that can efficiently detect Aβ plaques in the brain using imaging techniques (Chetelat et al., 2020), the level of soluble Aβo cannot yet be directly measured in the brain of live patients. Although we know that the level of soluble Aβo begin to increase in the brain ~10–15 years before any clinical symptoms of AD (Cline et al., 2018), it still need to be established if the hyperactivity observed in early AD patients is induced, at least partly, by this progressive accumulation of soluble Aβo in the brain as shown *in vitro* and in animal models. A way to bypass this limitation would be to investigate the level of Aβ_1−42_ and Aβ_1−40_ in the CSF or plasma of AD patients while measuring hippocampal hyperactivity by imaging techniques, although this method would only allow to investigate the global impact of Aβo on specific brain area dysfunctions.

## Hyperactivity in AD Animal Models

Neuronal hyperactivity has been detected in many transgenic AD mice such as the hAPP-J20, 3 × Tg-AD, APP23 × PS45, APP23, and APPswe/PS1D9 mice (Busche et al., 2008, 2012, 2015a; Rudinskiy et al., 2012; Sanchez et al., 2012; Maier et al., 2014; Nygaard et al., 2015) (Table 1). Using two-photon Ca^2+^ imaging, it was observed that 21% of cortical neurons displayed an increase of Ca^2+^ influx predominantly near the amyloid plaques in the APP23 × PS45 mouse model (Busche et al., 2008). A similar level of hyperactivity was also observed in the CA1 region of the hippocampus in young (1–2 months of age) APP23 × PS45 mice when Aβo begin to accumulate but no plaques are detected (Busche et al., 2012).

These results suggest that hyperactivity is an early pathological event that depends on the accumulation of Aβo rather than plaques *per se*, and that plaques might serve as a reservoir of toxic Aβo that amplify this excessive neuronal activity responsible, at least in part, for the marked synaptic and neuronal losses observed around plaques (Hefendehl et al., 2016). In parallel to this hyperactivity, another fraction (29%) of cortical neurons were also found to be hypoactive in 6–10 months old APP23 × PS45 mice when plaques are present (Busche et al., 2008). Since hypoactive neurones were only found after plaque formation, it is hypothesized that initial neuronal hyperactivity progressively switch to hypoactivity in AD (Busche and Konnerth, 2016), although the cellular and molecular mechanisms underpinning this shift still need to be determined.

To determine the direct implication of soluble Aβo on neuronal hyperactivation *in vivo*, exogenous Aβ species were also injected into the brain of wild-type mice. A single injection of Aβ-containing AD brain extracts and Aβ dimers were both found to induce a marked neuronal hyperactivity in CA1 neurons of wild-type mice (Busche et al., 2012; Zott et al., 2019). However, it should be noted that overexpression of additional APP fragments other than Aβo were also shown to induce hyperactivity and seizures in another mouse model (APP/TTA) (Born et al., 2014). Since it is difficult to tease apart the specific effects of each APP metabolites that are overexpressed in transgenic mouse models, the use of an animal model where fresh solutions of soluble Aβo are injected chronically into the hippocampus, such as the one we developed (Brouillette et al., 2012), could be advantageous to investigate the specific impact of Aβo on neuronal hyperactivity over time.

## Aβo-Induced Neuronal Hyperactivity *in vitro*

In line with these observations in AD mouse models, a myriad of studies performed *in vitro* also support the implication of Aβ on neuronal hyperexcitability using different types of Aβ solutions (Table 1). Indeed, application of the toxic Aβ peptide fragment consisting of amino acid residues 25 through 35 (Aβ_25−35_) to rat hippocampal cultures increased the intracellular levels of Ca^2+^ and the action potential activity in neurons (Brorson et al., 1995). Another study found that synthetic Aβ_1−42_ oligomers applied in primary neuronal cultures induced a dose-dependent decrease in neuronal viability that was cause, at least partly, by neuronal overexcitation (Sanchez-Mejia et al., 2008). Moreover, Aβ_1−42_ oligomers were found to induce aberrant neuronal activity in primary hippocampal neurons and in hippocampal slices from 3-month-old Tg2576 mice (Ciccone et al., 2019). Extracellular elevation of endogenously released human Aβ induced by inhibiting its degradation also rise up the synaptic vesicle release probability, and results in neuronal overexcitation in rat hippocampal cultures and in acute hippocampal slices (Abramov et al., 2009).

Similarly, higher levels of extracellular human Aβ_1−40_ monomers and dimers augmented synaptic vesicle release which in turn leads to hyperactivity of excitatory synapses in cultured hippocampal neurons and acute hippocampal slices (Fogel et al., 2014). More recently, it was shown that Aβ-containing AD brain extracts and purified cross-linked Aβ dimers were able to induce hyperactivity in active CA1 neurons treated with bicuculline in wild-type mouse hippocampal slices (Zott et al., 2019). Furthermore, increased Ca^2+^ transients and excessive neuronal excitability have been observed in neurons derived from human induced pluripotent stem cell (hiPSC) lines carrying familial AD mutations (Park et al., 2018; Ghatak et al., 2019).

## Cellular and Molecular Mechanisms Underpinning Aβo-Induced Neuronal Hyperactivity

**Figure 1:**
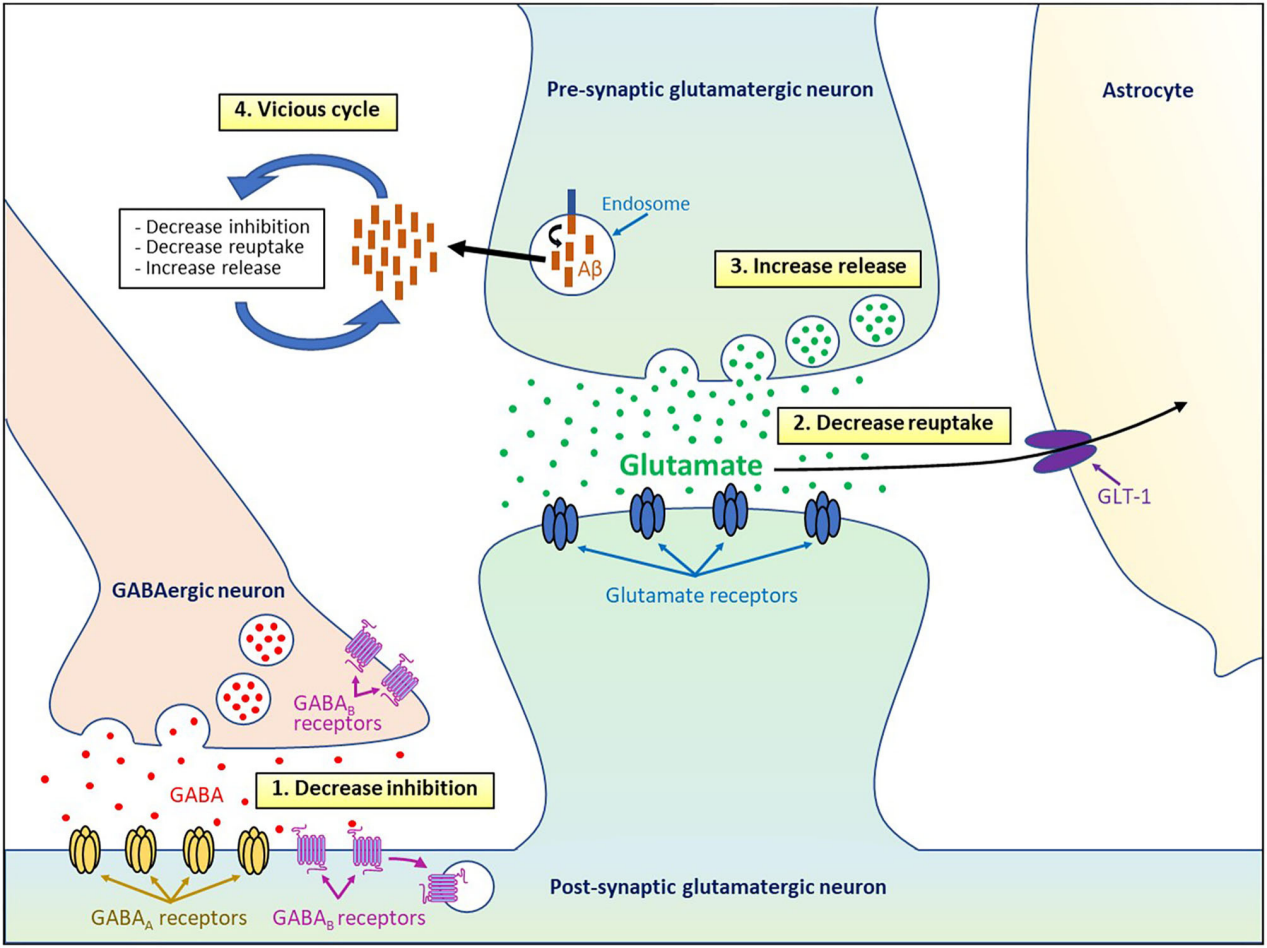
Cellular and molecular mechanisms underpinning Aβo-induced neuronal hyperactivity.

Different studies have revealed various cellular and molecular mechanisms to explain how Aβo might induce neuronal hyperactivity (Figure 1). Several lines of evidence obtained in AD mouse models suggest that soluble Aβo alter the excitation/inhibition balance by decreasing the inhibitory GABAergic function, which in turn induced an excessive activation of the excitatory glutamatergic system in AD mice (Busche et al., 2008; Palop and Mucke, 2010; Busche and Konnerth, 2016; Styr and Slutsky, 2018). Indeed, hyperactivity of the cortical neurons in APP23 × PS45 mice was linked to lower GABAergic inhibition instead of higher glutamatergic transmission, and the activity of the hyperactive neurons was found to be decreased by diazepam, a benzodiazepine that increase the probability of opening the γ-aminobutyric acid type A (GABA_A_) receptor channels (Busche et al., 2008).

These results are consistent with another study showing that GABA_A_ receptors localized in the temporal cortex of AD patients have a reduction of current, a higher rate of desensitization, and are less sensitive to GABA (Limon et al., 2012). Higher excitatory and lower inhibitory synaptic activities have also been reported in AD hiPSC-derived neurons (Ghatak et al., 2019). On the other hand, aberrant excitatory neuronal activity triggers by Aβ in the cortex and hippocampus of hAPP-J20 mice was found to induce subsequent maladaptive inhibitory mechanisms that reduce overexcitation (Palop et al., 2007), which could potentially be involved in the gradual switch to hypoactivity seen in animal models and AD patients (Celone et al., 2006; Persson et al., 2008; Sperling et al., 2009; Reiman et al., 2012; Busche and Konnerth, 2016).

Another mechanism that could explain hyperactivity generates by Aβo relies on the accumulation of glutamate at the synapse. Indeed, *in vivo* infusion of Aβ_1−42_ and Aβ_25−35_ into the rat cholinergic magnocellular nucleus basalis was shown to induce extracellular glutamate accumulation (Harkany et al., 2000). Fibrillar Aβ was also reported to decrease glutamate reuptake by both neuronal and glial cells (Harris et al., 1996; Parpura-Gill et al., 1997). More recently, it was found that Aβo-dependent hyperactivity in active CA1 neurons was triggered by impaired reuptake of synaptically released glutamate, which in turn potentiate excitatory glutamatergic transmission (Zott et al., 2019).

This reuptake suppression was shown to be induced by lower levels and membrane diffusion obstruction of the astroglial excitatory amino-acid transporter 2 (EAAT2; termed GLT-1 in mice) (Jacob et al., 2007; Hefendehl et al., 2016; Zott et al., 2019), a glutamate transporter that is predominant in the CA1 area and whose activity is reduced in the early stages of AD (Masliah et al., 1996; Hefendehl et al., 2016). In line with these observations, neuronal hyperexcitability observed in 5 × FAD mice was attenuated by increasing the expression of GLT-1 and by reducing changes in dendrite morphology, synaptic strength, and NMDA/AMPA receptors activity ratios after inhibiting the nuclear factor of activated T cells 4 (Sompol et al., 2017), a protein overactivated in the early stages of AD (Abdul et al., 2009).

An alternative mechanism by which Aβo may deregulate glutamate homeostasis implicates aberrant release of glutamate stored in pre-synaptic vesicles. Soluble Aβo have been shown to increase the release of pre-synaptic vesicles in hippocampal neuronal cultures, whereas the activation of inhibitory GABA_A_ receptors by the agonist taurine was able to block the accumulation of glutamate at the synaptic cleft (Brito-Moreira et al., 2011). Moreover, application of Aβ_1−42_ oligomers on hippocampal cultures was reported to increase the amount of synaptic vesicles and their exocytosis by disrupting the synaptophysin/VAMP2 complex at the pre-synaptic terminals (Russell et al., 2012). Even a small elevation of endogenous Aβ_40_ and Aβ_42_ peptides of different lengths and molecular conformations was able to accelerate the vesicle exocytosis rate and increased release probability of active neurons in hippocampal cultures (Abramov et al., 2009). Given that both higher and lower levels of endogenous extracellular Aβ oligomers reduced short-term facilitation of vesicle release (Abramov et al., 2009), these results indicate that the level of Aβ peptides needs to be tightly control to keep the vesicle release probability in the optimal range. Application of Aβ_40_ monomers or dimers was also shown to induce hyperactivity by augmenting vesicle release probability at excitatory synapses after promoting pre-synaptic CA^2+^ influx via APP homodimerization in hippocampal cultures and slices (Fogel et al., 2014). Interestingly, various Aβ peptides such as Aβ_1−42_, Aβ_1−40_, Aβ_1−28_, and Aβ_25−35_ were all found to increase potassium-evoked glutamate release from hippocampal slices in a dose-dependent manner (Kabogo et al., 2010).

Lower reuptake and higher release of glutamate can also act synergistically to increase the load of glutamate in the synaptic cleft and lead to its “spillover” to activate extrasynaptic GluN2B-containing NMDA receptors that were found to promote neuronal death (Parsons and Raymond, 2014). Interestingly, prolonged activation of NMDA receptors has been shown to induce endocytosis and lysosomal degradation of the post-synaptic GABA_B_ receptors (Terunuma et al., 2010), which could in turn amplify neuronal excitability by decreasing the inhibitory action of GABA in AD. Moreover, lower axonal trafficking and reduced expression of the pre-synaptic GABA_B_ receptors in AD were reported to increase Aβ formation (Dinamarca et al., 2019). Since neuronal and synaptic activity were shown to increase the production and secretion of Aβ (Cirrito et al., 2005; Dolev et al., 2013; Yamamoto et al., 2015), the hyperactivity induced by Aβo can also favor an excessive release of Aβ and consequently causes a vicious cycle that amplifies and perpetuates the deleterious effects of Aβo on cell function. Using a chemogenetic approach, it was reported that chronic attenuation of aberrant neuronal activity was able to reduce amyloid plaque formation and synapse loss (Yuan and Grutzendler, 2016).

## Impact of Aβo-Induced Hyperactivity on Cell Death

**Figure 2:**
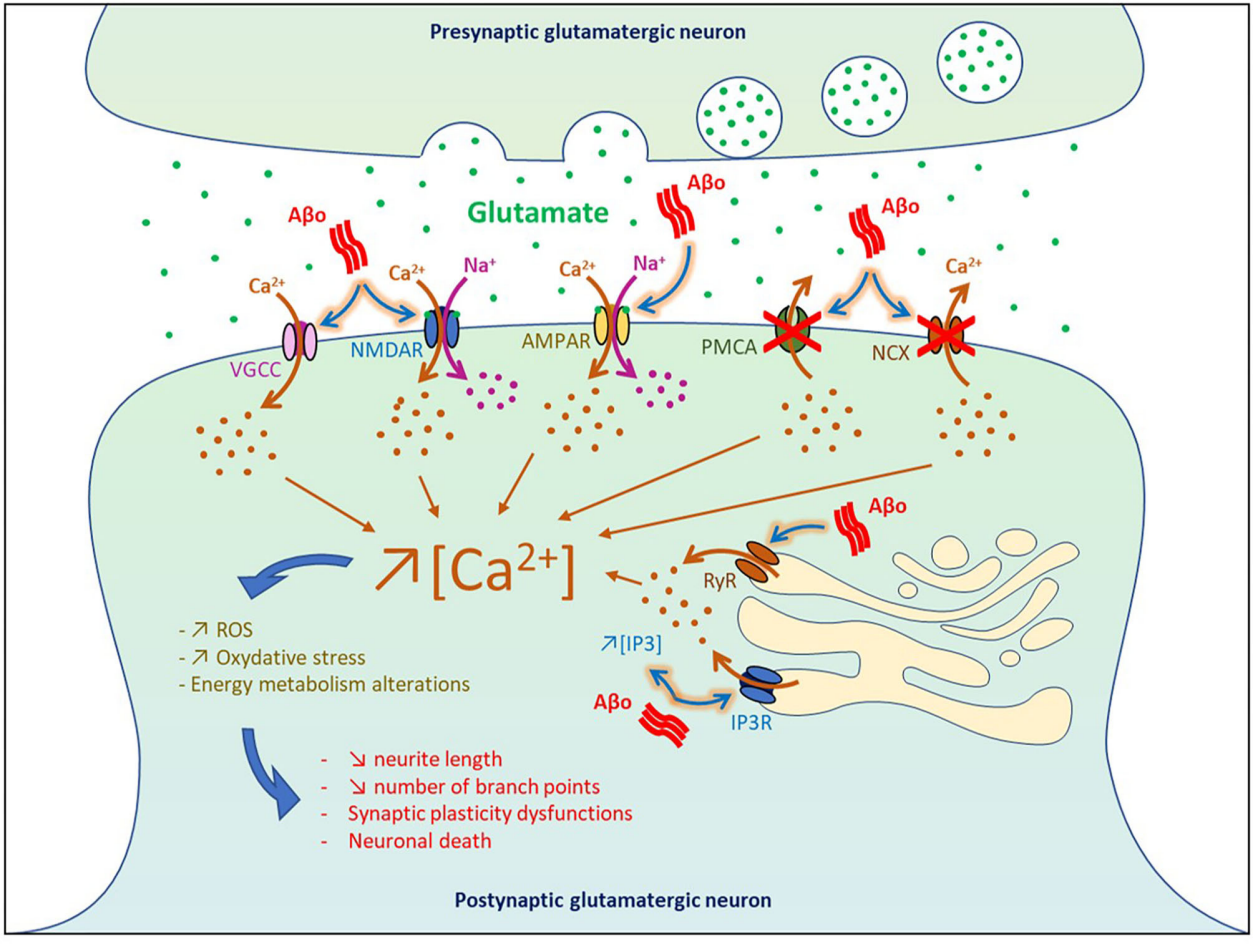
Impact of Aβo-induced hyperactivity on cell death. VGCC, voltage-gated Ca^2+^ channel; NMDAR, NMDA receptor; AMPAR, AMPA receptor; PMCA, plasma membrane calcium ATPase NCX, Na+/Ca2+ exchanger; IP3, inositol 1,4,5-trisphosphate; IP3R, IP3 receptor; RyR, ryanodine receptor.

By blocking glutamate reuptake and facilitating its pre-synaptic release, soluble Aβo increased glutamate concentration at the synaptic cleft and subsequently affect post-synaptic neurons by overactivating glutamatergic NMDA and AMPA receptors. It was shown that higher pre-synaptic release of glutamate induced by soluble Aβ generated a massive entry of Ca^2+^ and Na^+^ through NMDA receptors, which in turn impaired intracellular signaling pathways involved in synaptic plasticity and produced deleterious effects on neurons leading ultimately to cell death (Calvo-Rodriguez and Bacskai, 2020) (Figure 2). In physiological condition, Ca^2+^ concentration is finely balanced to maintain a lower level in the cytosol than in the extracellular space or some cell organelles such as the endoplasmic reticulum (ER) and lysosome. When this balance is disturbed in AD, overactivation of Ca^2+^-dependent intracellular pathways impaired energy metabolism, produced reactive oxygen species (ROS), and oxidative stress that eventually lead to cell death (Belkacemi and Ramassamy, 2012; Calvo-Rodriguez and Bacskai, 2020).

Using neuronal culture and entorhinal–hippocampal organotypic slices, it was found that Aβ_1−42_ oligomers dysregulated Ca^2+^ homeostasis and triggered neuronal death through both NMDA and AMPA receptors by generating ROS that derived in part from mitochondrial sources (De Felice et al., 2007; Wang and Zheng, 2019). *In vivo* infusion of Aβ_1−42_ and Aβ_25−35_ in the rat cholinergic magnocellular nucleus basalis induced a rapid accumulation of intracellular Ca^2+^ in the vicinity of the injection site followed by cell death 3 days post-injection (Harkany et al., 2000). In human cortical cell cultures, Aβ_1−38_ and Aβ_25−35_ increased the intracellular basal level of Ca^2+^ and amplified Ca^2+^ influx induced by excitatory amino acid (EAA), thereby potentiating EAA-induced neuronal degeneration (Mattson et al., 1992). Injection of Aβ-containing AD extracts in the CA1 area was also reported to reduce neurite length and the number of branch points in wild-type mice (Zott et al., 2019).

At the level of the plasma membrane, Aβo can also increase the intracellular levels of Ca^2+^ by (1) inhibiting the Ca^2+^-efflux ATPase or exchangers (Wu et al., 1997; Kim et al., 1999; Mata, 2018), and by (2) intensifying Ca^2+^ influx through L-type, T-type, and N-type voltage-gated Ca^2+^ channels (Ueda et al., 1997; Ekinci et al., 1999; MacManus et al., 2000; Thibault et al., 2012; Min et al., 2013). Moreover, Aβo were found to increase Ca^2+^ release from the ER to the cytosol by enhancing the function of ryanodine receptors and by increasing inositol 1,4,5-trisphosphate receptor (IP3) production and binding to its receptors (Cowburn et al., 1995; Shtifman et al., 2010; Demuro and Parker, 2013; SanMartin et al., 2017). These results are in line with the beneficial effects observed in some AD patients with the memantine compound, an antagonist of the NMDA receptors that reduced Ca^2+^ influx into cells (Robinson and Keating, 2006).

## Impact of Aβo-Induced Hyperactivity on Memory Performance

It is well-established that in healthy individuals hippocampal activity increased when performing different types of memory tasks such as spatial navigation, episodic and associative memory tasks (Sperling et al., 2003; Zeineh et al., 2003; Moser et al., 2017). This higher neuronal activity is essential to induce synaptic plasticity to encode and consolidate new information learned while executing the task. But what happen when the hippocampus gets overactivated? Excessive neuronal activity in the hippocampus was first observed in animal models of aging and has been shown to induce age-related memory deficits (Koh et al., 2010; Thome et al., 2016; Haberman et al., 2017). In AD, hippocampal hyperactivity can be detected in the preclinical et prodromal stages of the disease when memory deficits are still very subtle and can hardly be perceived by neurocognitive exams (Mondadori et al., 2006; Filippini et al., 2009; Bateman et al., 2012; Reiman et al., 2012). Indeed, before clinical symptoms become apparent in APOE4 carriers, higher hippocampal activation was associated with lower grid-cell like representation in the entorhinal cortex when performing a virtual spatial-memory task (Bookheimer et al., 2000; Kunz et al., 2015).

In APP knock-in mice with human APP containing three mutations, grid cells were shown to degenerate when Aβ depositions are emerging, and started to lose connection with place cells in the hippocampus when mice were getting old, which prevented the hippocampus to recreate spatial maps to distinguish between different environments (Jun et al., 2020). Memory deficits were also observed in APOE4 knock-in mice, in which the APOE gene is replaced by knocking in the human ε4 allele (Andrews-Zwilling et al., 2010). Transplantation of interneuron precursor cells and treatment with pentobarbital to promote the inhibitory action of GABA were both able to attenuate these cognitive dysfunctions in APOE4 knock-in mice (Andrews-Zwilling et al., 2010).

In presymptomatic individuals carrying the AD-associated PS1 E280A mutation, increased activation of the right anterior hippocampus was observed when performing a face-name associative encoding task (Quiroz et al., 2010). Hippocampal hyperactivity was even detected in elderly with Aβ plaque deposition who doesn't showed episodic memory impairment (Mormino et al., 2012), suggesting that Aβ-dependent hyperactivation is an early event that might be present before memory deficits become apparent in some hippocampal-dependent memory tasks. In cognitively normal elderly, higher hippocampal activation at baseline has been shown to be correlated with increased longitudinal Aβ plaque deposition and progressive memory decline across time (Leal et al., 2017).

In another study, the presence of Aβ plaques in the neocortex was associated with impaired episodic memory deficits in both asymptomatic elderly and MCI individuals (Pike et al., 2007). Amnestic MCI and non-demented older adults also showed aberrant activity in the dentate gyrus and CA3 regions of the hippocampus during a pattern-separation task, which markedly depends on the hippocampus (Yassa et al., 2011; Bakker et al., 2012, 2015). Moreover, MCI patients with Aβ plaque depositions were found to have more pronounce hippocampal activation at baseline and faster clinical progression compared to Aβ negative MCI elderly (Huijbers et al., 2015).

Transcranial magnetic stimulation (TMS) is a non-invasive form of brain stimulation technique that not only allow to monitor variations in intracortical inhibition and excitation but might also serve as a diagnostic tool and a way to modulate cortex activity to ameliorate memory function in AD patients. Indeed, repetitive TMS (rTMS) applied to the dorsolateral prefrontal cortex (DLPFC) has been shown to improve performance on an action naming memory task in mild AD as well as object naming in moderate to severe AD patients (Cotelli et al., 2006, 2008). A longer treatment (five times a week for 4 weeks) with rTMS over the left DLPFC was even able to enhance language performance of AD patients that lasted for 8 weeks after ending the stimulations (Cotelli et al., 2011). Moreover, high-frequency rTMS over the DLPFC improved memory performance in the mini-mental state examination (MMSE) in patients with mild to moderate AD, whereas high-frequency rTMS over the right inferior frontal gyrus increased attention and psychomotor speed of MCI and mild AD patients in the trail making test (Eliasova et al., 2014). Another study has found that application of rTMS for 6 weeks over the parietal P3/P4 and posterior temporal T5/T6 areas improved cognitive function in mild to moderate AD patients in three different neuropsychological tests (Zhao et al., 2017). Although we still don't know if rTMS can impact Aβ accumulation, this technique holds great promise to tackle neuronal hyperactivity and acts on it to improve cognitive performance of AD patients.

Neuronal hyperactivity also affects memory performance in various animal models. All the transgenic AD mouse models showing network hyperexcitability such as the hAPP-J20, 3 × Tg-AD, APP23 × PS45, APP23, Tg2576, and APPswe/PS1D9 mice, were found to have memory deficits in various memory tasks (Busche et al., 2008, 2012, 2015a; Rudinskiy et al., 2012; Sanchez et al., 2012; Maier et al., 2014; Nygaard et al., 2015). Direct injections of soluble Aβ_1−42_ oligomers into the hippocampus also induced memory deficits that were reversed by sequestering Aβo with transthyretin (Brouillette et al., 2012). Cognitive functions were also improved in hAPP-J20 and 3 × Tg-AD mice by suppressing neuronal overactivation with levetiracetam, an anti-epileptic drug that facilitate inhibitory GABAergic neurotransmission (Sanchez et al., 2012; Nygaard et al., 2015).

Memory plasticity can be modeled by inducing long-term potentiation (LTP) or long-term depression (LTD) in cell cultures or animal models (Nabavi et al., 2014). Nanomolar and micromolar levels of Aβ dimers and trimers were shown to inhibit LTP, increase LTD and reduce dendritic spine density in organotypic hippocampal slices (Townsend et al., 2006; Shankar et al., 2007, 2008; Li et al., 2009). It was found that Aβo altered LTP and LTD by decreasing neuronal glutamate reuptake, thereby contributing to the diffusion of glutamate outside the post-synaptic density where it can activate extrasynaptic GluN2B-containing NMDA receptors and induced cell death (Li et al., 2009, 2011; Hardingham and Bading, 2010). On the contrary, smaller (picomolar) concentration of Aβ_42_ was shown to enhance LTP and memory formation (Puzzo et al., 2008), suggesting that the level of Aβ needs to be finely tuned to prevent synaptic failure and ensuing cognitive impairment.

As the disease progresses and cognitive decline worsens, hippocampal activation decreased gradually at the basal level and when AD patients performed a task-related hippocampal activity (Dickerson et al., 2005; Pariente et al., 2005; Celone et al., 2006). In a prospective study it was found that MCI individuals shifted from hippocampal hyperexcitability to hypoactivation at the baseline level over time, and that deterioration of memory performance was associated with the rate of decrease in hippocampal activity (O'Brien et al., 2010). Collectively, these studies suggest that high neuronal activity induced, at least in part, by Aβ accumulation is a very early phenomenon in AD pathogenesis that has a deleterious impact on memory abilities.

## Impact of Aβo-Induced Hyperactivity on Epileptiform Activity

Since neuronal hyperactivation is characterized by an increase in frequency and amplitude of neuronal firing, it is not surprising to observe abnormal level of synchronization between excitatory glutamatergic neurons that fired together at the same time, which in turn increased the incidence of epileptiform activity and seizure observed in AD patients and AD animal models. Although the prevalence rates vary considerably between studies (1.5–64%) because of limitation and methodological issues to detect non-convulsive epileptiform activity, a rate of 64% has been observed in cohorts monitored carefully at all stages of AD (Friedman et al., 2012).

Interestingly, seizures have been shown to occur more frequently in younger AD patients (Vossel et al., 2013; Sherzai et al., 2014), when neuronal hyperactivity is more prominent. In patients with early-onset AD that developed the disease before 65 years old, seizures were detected in 45% of cases (Samson et al., 1996). A seizure rate of 28% was also observed in people with familial AD carrying mutations in *APP, PS1*, or *PS2* genes (Shea et al., 2016). In a prospective study of 8 years, seizures were observed in 84% of patients with Down's syndrome who developed AD because of the duplication of chromosome 21 that contains the *APP* gene (Lai and Williams, 1989). The higher and earlier accumulation of Aβ in familial cases of AD and Down's syndrome supports the notion that neuronal hyperactivation induced by soluble Aβo is involved in epileptogenic activity seen at the onset of AD.

In the more common form of sporadic AD, epileptiform activity might be more prominent than previously recognized. Indeed, it was first found that only 2% of AD patients had subclinical non-convulsive epileptiform activity when recording EEGs for 30 min in awake patients (Liedorp et al., 2010). However, a more recent study detected subclinical epileptiform activity in 42% of the cases (four times more often than in healthy controls), using 24h EEGs in combination with 1 h magnetoencephalography (MEG) (Vossel et al., 2016). Interestingly, 90% of epileptiform activity occurred during sleep, and AD patients with subclinical epileptiform activity showed a faster rate of cognitive decline (Vossel et al., 2016). Using intracranial recording, clinically silent hippocampal seizures and epileptiform spikes were also observed during sleep in two AD patients without a history or EEG evidence of seizures (Lam et al., 2017).

These results are in line with the manifestation of non-convulsive seizure activity and epileptiform spike discharges observed using EEGs in various AD transgenic models. Like in humans, most of the epilepsies seen in AD mice are non-convulsive, with the exception of mice overexpressing human APPswe and PS1ΔE9 which have recurrent motor seizures (Minkeviciene et al., 2009; Palop and Mucke, 2010; Um et al., 2012). In hAPP-J20 and APPswe/PS1ΔE9 mice, pathological elevation of Aβo has been shown to elicit hyperexcitability and spontaneous non-convulsive epileptic activity, including spikes and sharp waves, in cortical and hippocampal networks (Palop et al., 2007; Minkeviciene et al., 2009).

As in humans, spontaneous epileptiform discharges were found to arise mainly during resting periods in hAPPJ20 mice (Verret et al., 2012). Enhancing inhibitory GABA current by restoring the level of voltage-gated sodium channels subunit Nav1.1 was shown to reduce network hypersynchrony, memory deficits, and premature mortality in hAPP-J20 mice (Verret et al., 2012). Aβ_1−42_ oligomers were also found to up-regulate the level of Nav1.6 subtype, which contribute to neuronal hyperexcitability observed in primary hippocampal neurons and in hippocampal slices from 3-month-old Tg2576 mice (Ciccone et al., 2019). In APPswe/PS1ΔE9 mice, electrographic and motor seizures were prevented by deleting the cellular prion protein, which was shown to interact with Aβ and triggered dendritic spine loss (Um et al., 2012).

## Effect of Aβo-Induced Hyperactivity on Gamma Oscillations and Slow Wave Activity

Normal neuronal synchrony is critical to generate oscillatory rhythmic activities within a certain range that allow different brain regions to communicate efficiently together in function of the brain state. Brain rhythms are formed when neuronal ensembles depolarized (most often with firing) and hyperpolarized their membrane potentials together in synchronized repeating sequences (Buzsaki and Watson, 2012). Five widely recognized brain waves have been characterized in function of their frequencies; delta (1–4 Hz), theta (4–8 Hz), alpha (8–12 Hz), beta (12–30 Hz), and gamma (30–150 Hz) oscillations. Each brain waves have been associated with a particular brain state, where delta oscillations are more prominent during non-rapid eye movement (NREM) sleep whereas gamma oscillations are mostly detected when concentration is required, and tend to be localized to neuronal networks directly implicated in the task (Timofeev and Chauvette, 2017; Adaikkan and Tsai, 2020). For example, the amplitude (power) of gamma oscillatory activity was shown to be increased in the hippocampus during memory encoding and to predict effective memory formation in humans and mice (Jensen et al., 2007; Sederberg et al., 2007; Matsumoto et al., 2013; Yamamoto et al., 2014).

Given that Aβo-induced hyperactivity favors hypersynchrony, which in turn affects brain waves, one could expect that brain rhythms are altered at the onset of AD. In fact, gamma power has been shown to be reduced in MCI and AD patients (Herrmann and Demiralp, 2005; van Deursen et al., 2008), as well as in various AD mouse models (Verret et al., 2012; Goutagny et al., 2013; Iaccarino et al., 2016; Mably et al., 2017; Mondragon-Rodriguez et al., 2018). Interestingly, it was found recently that restoring slow gamma oscillation (40 Hz) in a non-invasive manner by simply exposing AD mice to 1 h of 40 Hz tons per day for a week was sufficient to reduce amyloid and tau pathologies not only in the auditory cortex but also in the hippocampus, to activate microglia, and to improve cognitive performance (Martorell et al., 2019). A stronger microglia response and a larger reduction of amyloid plaques were also found by combining auditory with visual stimulation to induce 40 Hz gamma waves (Martorell et al., 2019). Moreover, optogenetic stimulation of medial septal parvalbumin neurons at 40 Hz was reported to restore hippocampal slow gamma oscillations power and to ameliorate spatial memory in hAPP J20 mice (Etter et al., 2019).

Slow wave activity (SWA)—comprising slow oscillations (0.6–1 Hz) and delta waves—that is present during NREM sleep was also found to be disrupted in the early stages of AD (Lee et al., 2020). It is well-established that NREM sleep is particularly important to consolidate memories newly acquired during the awake state, and that SWA is critical to transfer novel information from the hippocampus to long-term memory storage across cortical areas (Steriade et al., 1993; Clemens et al., 2005; Diekelmann and Born, 2010). In individuals with MCI, lower delta and theta power during sleep was associated with declarative memory impairments, and more fragmentation of slow-wave sleep was observed relative to healthy elders (Hita-Yanez et al., 2012; Westerberg et al., 2012). It was shown that disruption of NREM SWA and deficits in hippocampus-dependent memory consolidation correlated with the level of Aβ plaque deposition in the medial prefrontal cortex of older adults (Mander et al., 2015). Cortical Aβ burden was also able to predict the lower amplitude of slow oscillations in elderly (Winer et al., 2019). Moreover, reduce slow-wave sleep was associated with higher level of Aβ in the plasma of MCI individuals (Sanchez-Espinosa et al., 2014). Interestingly, restoring slow oscillations by transcranial direct current stimulation was shown to improve memory performance in patients with early AD (Ladenbauer et al., 2017).

In line with these human studies showing the involvement of Aβ on SWA impairment at the onset of AD, disruption of SWA was also detected in mouse models of β-amyloidosis. SWA has been shown to be markedly disrupted in the hippocampus, neocortex and thalamus of APP23 × PS45 mice and in wild-type mice injected with synthetic Aβ (Busche et al., 2015b). Slow wave power was also decreased in young and older APPswe/PS1ΔE9 mice (Kastanenka et al., 2017, 2019). Moreover, both the APP/PS1 and Tg2576 mouse models exhibited an age-dependent decreased in delta and theta power (Kent et al., 2018), whereas 3 × Tg-AD mice showed slow waves at lower frequency (Castano-Prat et al., 2019). Remarkably, restoring slow oscillations using a GABA receptors agonist (benzodiazepine), a suppressor of Aβ production (β-secretase), or by optogenetic manipulation have all been shown to rescue memory deficits in various AD mouse models (Busche et al., 2015b; Kastanenka et al., 2017; Keskin et al., 2017).

## Treatments to Counteract Neuronal Hyperactivation in AD

Since Aβo-induced hyperactivity is an early pathological event that precedes plaque formation when soluble low-molecular-weight Aβo begin to accumulate in the human brain up to two decades before the symptomatic phase of the disease (Cline et al., 2018), acting on this detrimental phenomenon might prove beneficial to develop therapeutic approaches to prevent or at least slow down the disease progression. Since the excitation/inhibition balance has been shown to be compromised at the onset of AD primarily because of insufficient GABAergic inhibition (Busche et al., 2008; Palop and Mucke, 2010; Busche and Konnerth, 2016; Styr and Slutsky, 2018), using drugs that are capable of restoring the GABAergic system might potentially lower the hyperactivity triggers by Aβo and consequently AD pathogenesis.

The GABA_A_ receptors agonist taurine was found to attenuate neuronal hyperactivity by decreasing glutamate level released at the synapse (Brito-Moreira et al., 2011) (Table 2). Hyperactivity was also reduced in cortical neurons of APP23 × PS45 mice by increasing the inhibitory effect of GABA with diazepam (Busche et al., 2008). Topical application of GABA directly on the somatosensory cortex was reported to restore slow oscillations in APP mice (Kastanenka et al., 2017, 2019), whereas the topical application of the GABA_A_ agonist midazolam rescued the frequency and long-range coherence of slow waves in the frontal and occipital cortex of APP23 × PS45 mice and in wild-type mice infused with Aβo (Busche et al., 2015b). Moreover, intraperitoneal injection of the benzodiazepine clonazepam, which increase GABAergic function by acting on GABA_A_ receptors, has been shown to rescue slow waves and sleep-dependent memory consolidation in APP23 × PS45 mice (Busche et al., 2015b).

**Table 2:** Treatments to counteract neuronal hyperactivation in AD.

**Compounds**	**Types**	**Models**	**Effects**	**References**
Taurine	GABA_A_ receptors agonist	Aβ_1−42_ oligomers in hippocampal cultures	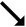 hyperactivity by 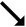 glutamate release	Brito-Moreira et al., 2011
Diazepam	Benzodiazepine	APP23 × PS45 mice	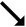 hyperactivity 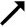 opening of GABA_A_ receptor channels	Busche et al., 2008
GABA	Neurotransmitter	APP mice	Restore slow oscillations	Kastanenka et al., 2017, 2019
Midazolam	Benzodiazepine	APP23 × PS45 mice, Aβo injected mice	Rescue the frequency and long-range coherence of slow waves	Busche et al., 2015b
Clonazepam	Benzodiazepine	APP23 × PS45 mice	Rescue slow waves and sleep-dependent memory consolidation	Busche et al., 2015b
GNE-0723	Modulator of NMDAR-GluN2A	hAPP-J20 mice	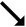 low-frequency oscillations, network hypersynchrony, and memory deficits	Hanson et al., 2020
NB-360	Inhibitor of β-secretase BACE	APP23 × PS45 mice	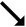 prefibrillary Aβ, hyperactivity, and memory deficits	Keskin et al., 2017
LY-411575	Inhibitor of γ-secretase	APP23 × PS45 mice	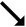 soluble Aβ levels, hyperactivity, and cognitive deficits	Busche et al., 2012
Levetiracetam	Anti-epileptic	hAPP mice	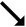 epileptiform activity, hyperactivity, hypersynchrony, DNA double-strand breaks; 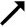 memory performance	Sanchez et al., 2012; Suberbielle et al., 2013, 2015; Nygaard et al., 2015
		Humans with MCI	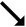 hyperactivity; 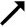 memory performance	Putcha et al., 2011; Bakker et al., 2012
Pyruvate and 3-β-hydroxybutyrate supplement	Dietary energy substrates	APPswe/PS1ΔE9 mice	Prevent energy metabolism deficits, hyperactivity, epileptiform activity	Zilberter et al., 2013
		Protofibrillar Aβ_1−42_ in hippocampal slices	Rescue network activity, synaptic function, LTP and energy metabolism	Zilberter et al., 2013

Recently, a positive allosteric modulator called GNE-0723 that can boost the activity of NMDAR containing GluN2A subunit contained in both excitatory pyramidal neurons and inhibitory interneurons has been tested in hAPP-J20 mice (Hanson et al., 2020). This compound was found to decrease aberrant low-frequency oscillations (12–20 Hz), network hypersynchrony, and memory deficits in hAPP-J20 mice, suggesting that this drug is able to reinstate the excitation/inhibition balance. Inhibitors of β-secretase BACE and γ-secretase, two enzymes involved in the production of Aβ, have also been shown to decrease Aβo-induced hyperactivity and cognitive impairments in APP23 × PS45 mice (Busche et al., 2012; Keskin et al., 2017). However, given the clinical trial failures obtained so far with these types of compounds, additional experiments are requested to develop more Aβ specific BACE and γ-secretase inhibitors and to find the appropriate doses and time of administration for an efficient therapeutic intervention.

Levetiracetam (Keppra) is an atypical anti-epileptic drug that is assumed to decrease impulse conduction across excitatory synapses by inhibiting pre-synaptic Ca^2+^ channels, and by acting on the synaptic vesicle protein SV2A (Lynch et al., 2004; Vogl et al., 2012). Interestingly, levetiracetam not only decreased epileptiform activity in hAPP mice, but also lower neuronal hyperactivation and hypersynchrony, improved memory performance, and reduced neuronal DNA double-strand breaks in AD mouse models (Sanchez et al., 2012; Suberbielle et al., 2013, 2015; Nygaard et al., 2015). In MCI individuals, treatment with a low dose of levetiracetam for two weeks was found to attenuate hippocampal hyperactivation and to ameliorate performance in a pattern-separation memory task (Putcha et al., 2011; Bakker et al., 2012).

Since higher level of glucose is required to provide the increase of energy associated with neuronal hyperexcitability, several cellular energy deficiencies have also been detected at the onset of AD (Velliquette et al., 2005; Guglielmotto et al., 2009; Avila et al., 2010). To compensate for this neuronal energy supply deficiency, an energy substrate-enriched diet (standard diet supplemented with pyruvate and 3-β-hydroxybutyrate) was administered for 5 weeks to APPswe/PS1ΔE9 mice (Zilberter et al., 2013). By restoring the level of glycogen in the brain of these AD mice, this treatment was able to prevent energy metabolism deficits, neuronal hyperexcitability, and epileptiform activity. Moreover, alterations in network activity, synaptic function, LTP, and energy metabolism induced by protofibrillar Aβ_1−42_ in hippocampal slices were rescued by using artificial cerebrospinal fluid supplemented with pyruvate and 3-β-hydroxybutyrate (Zilberter et al., 2013).

## Conclusion

A myriad of studies performed in humans, cell cultures, hiPSC lines carrying familial AD mutations, AD mouse models, and wild-type mice injected with soluble Aβo indicate that neuronal hyperactivity is an early detrimental event in AD pathogenesis. Multiple lines of evidence strongly suggest that the accumulation of soluble low-molecular-weight Aβo plays a major role in neuronal hyperexcitability observed at the onset of AD, although other factors might also contribute, such as tau, other APP metabolites, APOE4, glial responses, neuroinflammation, and oxidative stress. Encouragingly, a growing body of evidence indicates that neuronal hyperactivity may be potentially reversed, which could prevent cell death, improve cognitive impairments, decrease epileptiform activity, restore gamma oscillations, and slow wave activity. Decreasing the abnormal accumulation of soluble Aβo to avoid an excess of glutamate at the synaptic cleft and re-establishing the balance between synaptic excitation and inhibition might prove useful to ameliorate memory performance in the early stages of AD and prevent, or at least slow down, the neurodegenerative process that progressively takes place in the course of AD.

## Author Contributions

AH and JB wrote and approved the final manuscript. All authors contributed to the article and approved the submitted version.

## Conflict of Interest

The authors declare that the research was conducted in the absence of any commercial or financial relationships that could be construed as a potential conflict of interest.
